# Evaluation of Various Interventions to Valorize Dry-Aged Waste Products in Ground Beef Formulations

**DOI:** 10.3390/foods15111853

**Published:** 2026-05-23

**Authors:** Peyton S. Arnold, Cameron C. Catrett, Palika Dias-Morse, Jennifer C. Acuff, Derico Setyabrata

**Affiliations:** 1 Department of Animal Science, University of Arkansas, Fayetteville, AR 72701, USA; peytona@uark.edu (P.S.A.); ccatrett@uark.edu (C.C.C.); 2 Department of Animal Science, University of Arkansas Division of Agriculture, Fayetteville, AR 72701, USA; pdias@uark.edu; 3 Department of Food Science, University of Arkansas, Fayetteville, AR 72704, USA; jcacuff@uark.edu; 4 Department of Food Science, Center for Food Safety, University of Arkansas Division of Agriculture, Fayetteville, AR 72704, USA

**Keywords:** dry-aging, dry-aged crust, ground beef, valorization, meat waste

## Abstract

This study evaluated the impact of treated dry-aged crust inclusions on final ground beef quality. Ground beef (80 lean: 20 fat) was divided into: CON (beef only), NTC (non-treated crust), WW (warm-water-washed crust), DH (dehydrated crust), and SV (sous-vide crust). Treated crusts were chopped, mixed with ground beef (10% inclusion), reground, formed into patties, and subjected to quality and microbial analyses. The pH for day 1 (d1) samples was lower than for day 7 (d7) samples regardless of treatment (*p* < 0.05). No differences were found for proximate analysis, cook loss, or display loss (*p* > 0.05). An interaction effect was observed for all color traits (*p* < 0.05), demonstrating rapid color decline during display in both NTC and WW treatments compared to other treatments. Greater lipid oxidation was observed in CON compared to other treatments before and after display (*p* < 0.05). The CON, DH, and SV treatments had lower microbial concentrations than NTC and WW (*p* < 0.05). Texture profile analysis showed elevated hardness values in SV compared to CON, NTC, and WW, while DH did not differ from any treatment (*p* < 0.05). Our results indicate that DH and SV interventions minimally impact product quality while reducing initial microbial concentrations, suggesting potential use as intervention methods for dry-aged crust.

## 1. Introduction

Consumers desire an enjoyable eating experience, especially when it comes to meat products. Postmortem aging is a widely accepted natural process that can improve meat quality attributes such as tenderness, juiciness, and flavor [1]. In particular, the dry-aging process has been reported to greatly enhance meat flavor, generating unique flavors such as “beefy,” “brown-roasted,” and “nutty,” which are highly desirable to consumers and further increase demand [2,3].

During the dry-aging process, meat products are exposed to a controlled refrigerated environment without any packaging. Due to the lack of protective barriers, excessive moisture loss will occur, leading to the formation of a crust (dehydrated surfaces) that requires trimming to obtain the consumable portion.

Studies have reported that evaporative and trim losses following dry-aging can range from 15 to 50%, making the process highly costly [4,5,6,7,8]. Recent reports have indicated that dry-aged crust could be upcycled to enhance product functionality and flavor. A study by Xue et al. [9] reported that dry-aged crust inclusion in meat emulsion and ground beef generated a product with higher antioxidative properties and emulsifying capacity, while also increasing the brown-roasted flavor observed in the final product. Similarly, Park et al. [10] also identified that the crust can act as a flavor enhancer for ground beef patties and may also have antioxidant capacity.

Although recent studies have indicated the potential benefits of the crust as a functional ingredient, the utilization of the crust remains limited due to microbial contamination concerns from environmental exposure during the aging process. Recent studies reported that aerobic microbial concentration from the surface of dry-aged meat could range from 4 to 6 Log_10_ CFU/cm^2^ [6,8,11]. While some reports indicated a reduction in pathogenic bacteria (e.g., *Salmonella* spp., *E. coli* O157:H7, *Listeria monocytogenes*) concentration following dry aging [12,13], other studies also indicated that the extent of pathogen reduction was dependent on environmental conditions [14,15]. This indicated that both spoilage and pathogenic microorganisms could grow and persist on the dry-aged crust, necessitating interventions to mitigate microbial concerns prior to its use as a functional ingredient.

Currently, only limited information is available on potential interventions to mitigate microbial concerns in dry-aged crust. A recent investigation into the application of interventions to dry-aged crust suggests a reduction in microbial concentration following high-pressure processing [16]. However, the technology is mainly available to large-scale food manufacturers due to its high equipment costs and high-volume requirements, limiting its potential application to small-scale producers commonly associated with dry-aged production. Our recent parallel study evaluated six different low-cost and simple intervention techniques (acid wash, warm water wash, cold water wash, dehydration, sous vide, and UV light) to reduce microbial presence in dry-aged crust [17]. The authors found that dehydration and sous vide treatment of the crust significantly reduced the presence of *E. coli* O157:H7, *Salmonella* Heidelberg, and *Listeria monocytogenes*, indicating potential application of the techniques as an intervention step to minimize microbial concern from dry-aged crust. However, the impact of those interventions on final meat quality and palatability attributes remains unclear. Therefore, this study aims to evaluate low-cost interventions on the dry-aged crust and to assess the effect of crust inclusion on both quality and textural properties of ground beef patties. We hypothesize that processing interventions will improve microbial quality while minimally impacting patty quality.

## 2. Materials and Methods

### 2.1. Dry-Aged Crust Sample Preparation

Dry-aged crust trim was obtained from a commercial dry-aging processing facility. The crust sample was collected from beef striploins that were dry-aged for 14 days at 3 °C and 90% relative humidity. The crusts were shipped overnight in a plastic bag placed in a Styrofoam box with gel packs to the University of Arkansas Red Meat Laboratory. Upon arrival, crust samples were vacuum packaged and kept frozen until use (−80 °C). The frozen crust was thawed at 2 °C, divided into four groups, and assigned to the following treatments: non-treated crust (NTC), warm water wash (WW), dehydrated (DH), and sous-vide (SV). The WW treatment was selected for its low cost and accessibility for processors, and the DH and SV treatments were chosen based on the pathogen reduction efficiency reported by Sananikone et al. [17]. The WW crust was washed with 50 °C water for one minute, then allowed to drip for one minute. The DH crust was placed into a dehydrator (Pioneer 5-Tray Food Dehydrator; Cosori, Tustin, CA, USA) set to 60 °C for six hours. The SV crust was placed in a single layer in a vacuum bag, vacuum-packaged (Proxmax Vac. Table Top Vacuum Chamber Sealer; Promarksvac, Ontario, CA, USA), and then placed in a hot water bath (SmartVide sous-vide cooker; Sammic, Evanston, IL, USA) at 60 °C for 2 h. Following treatment, all treated crusts were vacuum-packaged and frozen until further processing (−80 °C). The crust samples were thawed (2 °C) 24 h before use and chopped in a food processor (Premier Series 11-Cup Food Processor; Cuisinart, Stamford, CT, USA) immediately prior to inclusion.

### 2.2. Beef Patty Preparation

A total of 3 independent batches were prepared in the current study. For each batch, ground beef patties were made from ground beef chubs (80 lean: 20 fat) combined with the previously treated crust. The chubs were equally divided into 5 groups and randomly assigned to 5 treatments: control (CON, no crust inclusion), NTC-, WW-, DH-, or SV-treated crust inclusion. The crust treatments were formulated with a 10% replacement of ground beef with the respective treated crust. The ground beef mixture was then mixed in a stand mixer (NSF Certified Commercial Series 8 Quart Stand Mixer; KitchenAid, Benton Harbor, MI, USA) for one minute at speed 4. Following mixing, each treatment was reground in a meat grinder for uniformity (4.5 mm plate; AE-G12N Meat Grinder; American Eagle, Chicago, IL, USA) and formed into at least 4 patties (125 g) for quality and textural analyses. The first patty was assigned for cook loss and texture profile analysis. The second patty was used for proximate analysis and d1 biochemical analyses. The third patty was designated for d1 microbial analysis and pH. The last patty was assigned for 7-day simulated color display, display loss, d7 microbial analysis and d7 biochemical analyses. The first and second patties were individually vacuum-packaged and kept frozen at −80 °C until analysis. The third and fourth patties were immediately used. The displayed patties were individually vacuum-packed and kept frozen at −80 °C following all analyses except biochemical analysis. The same prepared crust samples were utilized in all three independent batches.

### 2.3. pH

Measurements were taken with a benchtop meter (VWR pHenomenal IS 2100 L; Radnor, PA, USA) before and after the simulated display. The pH meter was calibrated using pH 4, 7, and 10 solutions. Three grams of each ground beef sample and 27 mL of deionized water were homogenized (T-25 Ultra Turrax, IKA Works Inc., Wilmington, NC, USA) at 8000 rpm for 15 s. The pH measurements were conducted in duplicate, recorded, and averaged.

### 2.4. Proximate Analysis

Two grams of d1 sample were weighed into aluminum dishes, placed in a 100 °C oven (1370GM Signature Gravity Convection Oven; VWR International, Radnor, PA, USA) for 24 h, then placed in a desiccator for 1 h before the final weight was taken. Moisture was calculated using the following equation: $\left(1-\frac{\left(Oven\;wt.\;-\;Pan\;wt.\right)}{Sample\;wt.}\right)\cdot 100\%$. Samples were then placed in a furnace overnight to calculate ash content. Ash was determined using the following equation: (Ash & Pan wt. − Pan wt.). Nitrogen was determined by the combustion method (AOAC 992.15-1992 (1996)) [18] using an ECS 8020 (ECS 8020—CHNS-O Analyzer; NC Technologies, Milan, Italy). Crude protein was calculated by the following equation: % nitrogen × 6.25. Fat percentages were calculated by difference: (100 − (Moisture +Protein + Ash).

### 2.5. Water Holding Capacity

Ground beef patties assigned for display loss were weighed (g) on d1, then packaged in polyvinyl chloride film (PVC; AEP Industries Inc., South Hackensack, NJ, USA) with an oxygen transmission rate of 1450 cm^−3^ · 645.2 cm^−2^ · 24 h^−1^ on a white 2S foam tray with a Dri-Loc 50 absorbent pad (Cryovac Sealed Air Corp., Duncan, SC, USA). Samples were subjected to a 7d simulated retail display, then removed after the display period. Patties were weighed on d7, and display loss was calculated using the following equation: [{(d1 wt. − d7 wt.)/(d1 wt.)} × 100].

To analyze cook loss, previously frozen patties (d1) were thawed (2 °C) 24 h before analysis. Patties were dried with a paper towel to remove excess moisture and weighed. Samples were cooked on a clamshell grill (149 °C) (Griddler GR-150; Cuisinart, Glendale, AZ, USA) until they reached an internal temperature of 69 °C, measured using a thermocouple. Samples were removed from the grill, placed on a paper towel, and allowed to rest for 10 min. Patties were blotted dry with a paper towel, then weighed to obtain the final cooked weight. Cook loss was calculated using the following equation: [{(raw wt. − cooked wt.)/(raw wt.)} × 100].

### 2.6. Microbial Analysis

Microbial analysis was performed on d1 and d7 to assess aerobic plate counts (APC), lactic acid bacteria (LAB), and yeast/mold (YM) concentrations before and after simulated display. A total of 20 g of sample was combined in a stomacher bag (WhirlPak; Madison, WI, USA) with 60 mL of 0.1% peptone water (BD Difco™; Sparks, MD, USA) and stomached (Stomacher 400; Seward, West Sussex, UK) at 260 rpm for 60 s. The rinsate was filtered out of the stomacher bag into a sterile tube and serially diluted (1:10). Samples were plated in duplicate to determine APC (Plate Count Agar; BD Difco™, Sparks, MD, USA), LAB (DeMan, Rogosa, and Sharpe Agar; HiMedia Laboratories LLC., Kennett Square, PA, USA), and YM (Yeast/mold cassettes; CompactDry™, Hardy Diagnostics, Santa Maria, CA, USA). APC plates were incubated at 38 °C for 48 h; LAB plates were incubated in an anaerobic environment at 35 °C for 72 h, and YM plates were incubated at room temperature (22 °C) for 72 h. Colonies were counted between detectable limits (25–350 for APC and LAB; 1–300 for YM), logarithmically altered, and expressed in log 10 CFU/mL rinsate.

### 2.7. Simulated Retail Display and Color Evaluation

Patties assigned for color evaluation were weighed (g), then packaged in polyvinyl chloride film (PVC; AEP Industries Inc., South Hackensack, NJ, USA) with an oxygen transmission rate of 1450 cm^−3^ · 645.2 cm^−2^ · 24 h^−1^ on a white 2S foam tray with a Dri-Loc 50 absorbent pad (Cryovac Sealed Air Corp., Duncan, SC, USA). Samples were then subjected to a 7d simulated retail display in an open-front multi-deck case with LED light bars (P105998A, Lux 1628–2040, 3500 K, 48″, 4.80 Watt., Hillphoenix Inc., Conyers, GA, USA) (2 °C) with daily location rotation and color measurement. On d7, patties were removed and used for further analysis.

Three color measurements were taken on the surface of each sample using a MiniScan (Model 4500; HunterLab; Reston, VA, USA) using Illuminant A, 31.8 mm aperture, and 10° observer to determine Commission Internationale de l’Eclairage (International Commission on Illumination) L*, a*, and b* values. Prior to the measurements being taken, the MiniScan was calibrated using black and white tiles, according to the manufacturer’s recommendations. Hue angle and chroma values were calculated from the L*, a*, and b* values using the following equations: hue angle = arctangent (b*/a*); chroma = (a*^2^ + b*^2^) ½ [19].

### 2.8. Thiobarbituric Acid Reactive Substances

Methods used in Thiobarbituric Acid Reactive Substances (TBARS) analysis were performed using those described in Setyabrata et al. [20] with minor modifications. Five grams of sample, 15 g of deionized water, and 50 µL of butylated hydroxyanisole were homogenized for 15 s. Then, 1 mL of homogenate was transferred into a 15 mL tube and combined with 2 mL of 20 mM 2-thiobarbituric acid/15% trichloroacetic acid reagent, then vortexed. Tubes were placed in a hot water bath (80 °C) for 15 min, then transferred into an ice water bath. After 10 min, samples were vortexed and centrifuged at 2000× *g* at 25 °C for 10 min. After being removed from the centrifuge, samples were filtered into a new 15 mL test tube through Whatman filter paper #4. A total of 200 µL of sample was transferred into a 96-well plate, and absorbance was read at 531 nm on a SynergyLX Microplate Spectrophotometer (BioTek Instrument Inc., Winooski, VT, USA). Final values were reported as mg malondialdehyde/kg of meat.

### 2.9. Texture Profile Analysis

Patties previously utilized for the cook loss evaluation were used for the texture profile analysis (TPA). After cooking and resting, samples were wrapped in aluminum foil and placed at 2 °C for 24 h. Four cores (2.54 cm diameter) were manually taken from each sample, and measurements were taken using a texture analyzer (TA.XTPlus Connect; Texture Technologies, Hamilton, MA, USA). Values for hardness, adhesiveness, resilience, cohesion, springiness, and chewiness were recorded and averaged for each attribute.

### 2.10. Statistical Analysis

Data were fit using a mixed model and analyzed using an ANOVA procedure in RStudio (Version 2026.04.0+526) (Boston, MA, USA), with patty as the observational unit. The study was a randomized complete block design (RCBD) with batch serving as the block. Data for pH, microbial enumeration, and TBARS were analyzed as a RCBD with a 5 × 2 factorial arrangement and the fixed effects of treatment, display day, and their interaction. For proximate analysis, water-holding capacity, and TPA, data was analyzed as a RCBD with treatment serving as the fixed effect. Instrumental color data were analyzed as a RCBD with repeated measures, with display day serving as the repeated measure, compound symmetry as the covariance structure, and the fixed effects of treatment, display day, and their interaction. Pairwise comparisons between the least-square means were computed using the lme4 and emmeans packages in RStudio (Version 2026.04.0+526). Differences were considered statistically significant at *p* < 0.05, with tendencies between 0.05 < *p* < 0.10.

## 3. Results and Discussion

### 3.1. pH

**Figure 1:**
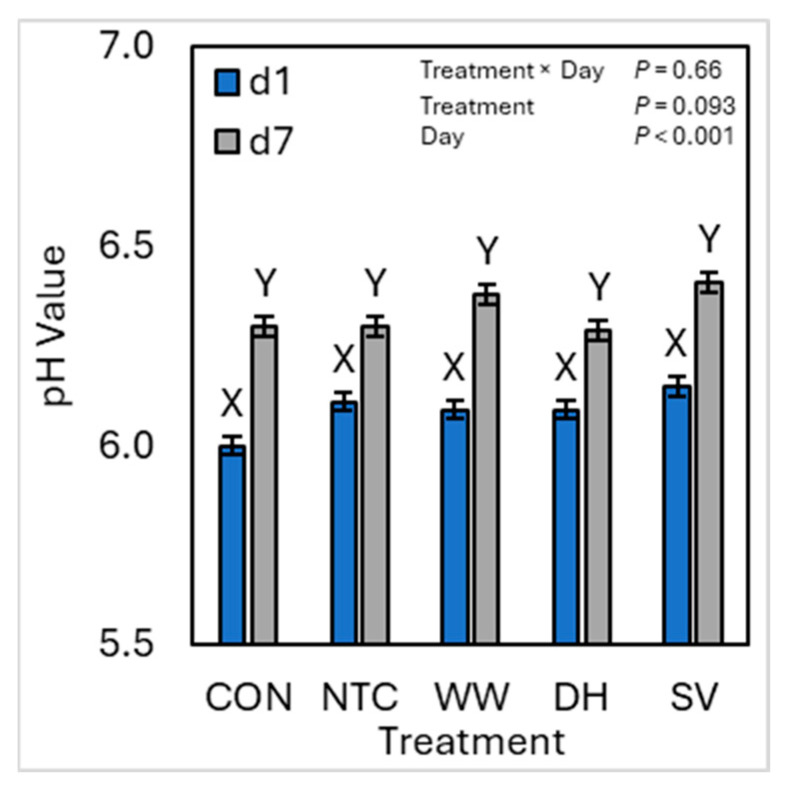
pH values of ground beef patties with differently treated dry-aged crust inclusions. ^XY^ Different superscript letters indicated a significant day effect (*p* < 0.05). Error bars represent the standard error of means.

The only significant day effect was observed for pH, with d1 samples having lower pH than d7 samples (*p* < 0.05; Figure 1). However, a tendency was identified in which the addition of the treated crust increased the pH of the samples compared to CON (*p* = 0.09). Previous studies evaluating the impact of dry-aged crust inclusion reported inconsistent impacts on the final pH of the product. Lee et al. [5] found that the inclusion of dry-aged beef crust decreased the pH of pork patties. Conversely, Park et al. [21] and Setyabrata et al. [22] reported an increase in pH when dry-aged crust or trim was added to the product. These conflicting observations indicated that the pH alteration likely depends on the pH of the added crust. In the current study, the added crust had pH values of 6.61, 6.55, 6.54, and 6.89 for NTC, WW, DH, and SV, respectively, explaining the increasing pH trend.

### 3.2. Proximate Composition

No significant differences were found in the moisture, ash, protein, or fat content of the samples (*p* > 0.05; Table 1). Regardless of the crust preparation method, the final ground beef products had comparable compositions. The observed results, however, differ from those reported by Xue et al. [9], who reported decreased moisture and ash with the addition of crust. The discrepancy could be attributed to differences in crust preparation methods between the studies, with the previous study using lyophilization. Due to the nature of the process, lyophilization tends to remove more moisture than air dehydration. Additionally, most of the methods used in the current study did not remove any moisture and, therefore, minimal alterations to the final product composition were expected.

### 3.3. Water Holding Capacity

No significant differences were found among display loss values, indicating similar loss across all treatments (*p* = 0.484; Table 1). Similarly, no differences were found between treatments for cook loss (*p* = 0.161; Table 1).

The currently observed results differ from previous investigations. Previous studies reported an improvement in water holding capacity with the addition of dry-aged beef crust, leading to lower cook loss and greater yield in ground meat products [5,9] and emulsion sausages [23]. Furthermore, both Lee et al. [5] and Lee et al. [23] found that greater water-holding capacity was observed with higher dry-aged crust inclusion. It is worth noting that although not statistically significant, the ground beef patties with treated crust inclusions in the current study displayed lower cook loss values (ranging from 3.7 to 4.9%) compared to CON. However, no clear trend was observed for display loss in the current study, which conflicts with the previous report by Xue et al. [9], in which the authors found lower display values in patties with crust inclusion.

As mentioned in the previous discussion, the observed difference in water-holding capacity might be attributed to the form of the crust when incorporated into the product. The studies by Xue et al. [9], Lee et al. [23], and Lee et al. [5] utilized lyophilized dry-aged crust in their experiments. Lee et al. [5] suggested that the lyophilized crust may form stronger bonds with water molecules, allowing it to retain moisture more effectively. The same authors also suggested that the lyophilization process enabled the final dried crust to have a high rehydration potential, thus making it capable of absorbing any moisture released from the meat and limiting moisture loss. Compared with the current study, most of the selected treatments were less likely to cause extensive dehydration caused by the lyophilization process. The treated dry-aged crust used in the current study likely still contained a high moisture content and had low rehydration potential. This could explain the minimal alteration in the overall moisture content of the ground beef patties and corroborates the reported proximate composition results. However, given the trend toward lower cook loss with crust inclusion, it could be hypothesized that greater inclusion of non-lyophilized crust could still increase the water-holding capacity of the final products, although further study would be required to confirm the hypothesis.

### 3.4. Microbial Analysis

**Figure 2:**
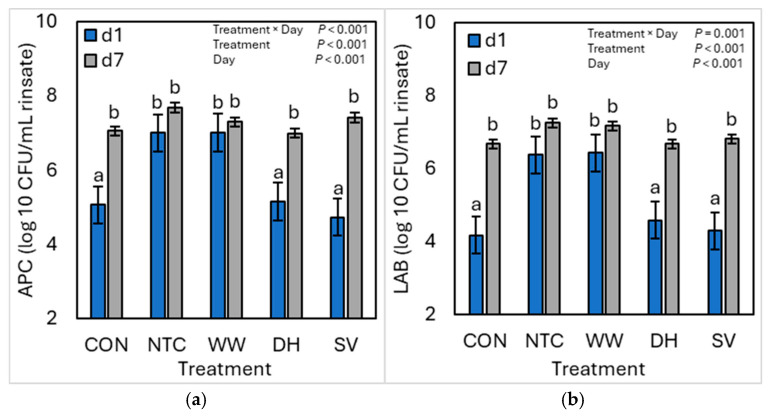
Aerobic bacteria (APC; (**a**)) and lactic acid bacteria (LAB; (**b**)) concentration of ground beef patties with treated dry-aged crust inclusions. ^ab^ Different superscript letters indicated a significant Treatment × Day interaction (*p* < 0.05). Error bars represent the standard error of means.

Both APC (Figure 2a) and LAB (Figure 2b) had a significant treatment × day interaction effect (*p* < 0.05). For both APC and LAB counts, CON, DH, and SV samples were observed to have lower microbial counts than NTC and WW on d1. However, all treatments did not differ from each other on d7. Both NTC and WW microbial counts were not different between d1 and d7.

**Figure 3:**
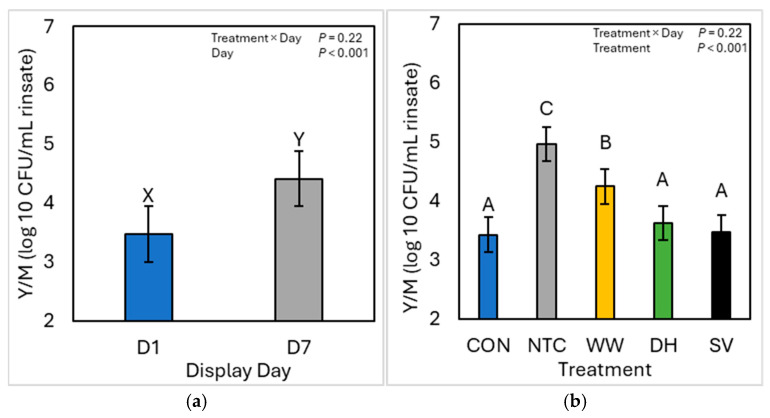
Yeast/mold concentration of ground beef patties with treated dry-aged crust inclusions. (**a**) day effect, (**b**) treatment effect. ^XY^ Different superscript letters indicated a significant day effect (*p* < 0.05). ^A–C^ Different superscript letters indicated a significant treatment effect (*p* < 0.05). Error bars represent the standard error of means.

The YM counts showed a significant day (*p* < 0.05; Figure 3a) and treatment effect (*p* < 0.05; Figure 3b). No significant interaction effect was observed for YM concentration (*p* = 0.215). As expected, the concentration of YM increased from d1 to d7 (*p* < 0.05), regardless of treatment. Among the observed treatments, lower YM counts were observed in CON, DH, and SV, with WW as an intermediate, and NTC had the highest YM count (*p* < 0.05).

Microorganisms have been suggested to play a critical role in the dry aging process, as their activity might contribute to the palatability improvement observed in dry-aged meat [4,24,25,26]. Microorganisms such as *Thamnidium*, *Mucor*, and *Debaryomyces hansenii* have been reported to release proteases and produce collagenolytic enzymes that improve the tenderness and flavor of the final dry-aged products [27,28,29]. Recent studies have also evaluated the potential inoculation during the dry-aging process to accelerate and enhance the final dry-aged product [30,31]. However, this heightens concerns about dry-aged crust utilization, as microbial contamination could lead to safety and spoilage issues. In mitigating this issue, several approaches have been evaluated as interventions during the dry-aging process, such as UV light application [8,25], dry-aging permeable bags [32,33] and coating application [34,35]. While reducing the microbial concentrations on the surface, the microorganisms were not completely eliminated and may pose a safety and quality threat.

Currently, little information is available on microbial intervention evaluation of dry-aged crust and the microbial quality of final meat products following the inclusion of dry-aged crust. Park et al. [10] reported that the inclusion of 5% lyophilized crust significantly increased the total aerobic bacteria concentration of beef patties compared to a beef-only control, which is not surprising, as lyophilization could preserve microorganisms’ viability [36]. A recent study by Witte et al. [16] explored the utilization of high-pressure processing as an intervention to reduce microbial concentration in dry-aged crust prior to incorporation into raw fermented sausages. The authors reported a significant reduction in microbial concentration in the dry-aged crust, and comparable microbial concentrations between the control and the sample with dry-aged crust addition were measured throughout the ripening day, suggesting a potential intervention for dry-aged crust.

Based on the results of the current study, DH and SV treatments may serve as effective interventions to minimize spoilage concerns associated with the utilization of dry-aged crust. Regarding pathogen mitigation, our recent parallel study [17] evaluated various intervention methods that could be applied to potentially reduce *E. coli* O157:H7, *Salmonella* Heidelberg, and *Listeria monocytogenes* contamination on the crust. The authors observed that dehydration and sous vide treatments resulted in a 5-log reduction in *E. coli* O157:H7 and *Salmonella* Heidelberg. These results align with the current study, indicating that DH and SV had lower microbial concentrations than NTC and WW, and were comparable to CON. However, further targeted challenge studies are required to fully validate the microbiological safety of including dry-aged crust in final formulations. Compared with NTC and WW, both DH and SV treatments may have been more effective because the heat application during the treatment significantly reduced microbial survivability. It has been reported that a spoilage concentration of 10^7^–10^8^ CFUs is deemed unacceptable for consumption [37]. All treatments except d7 NTC and WW were below this threshold, while d7 NTC and WW were within this range.

### 3.5. Instrumental Color

**Figure 4:**
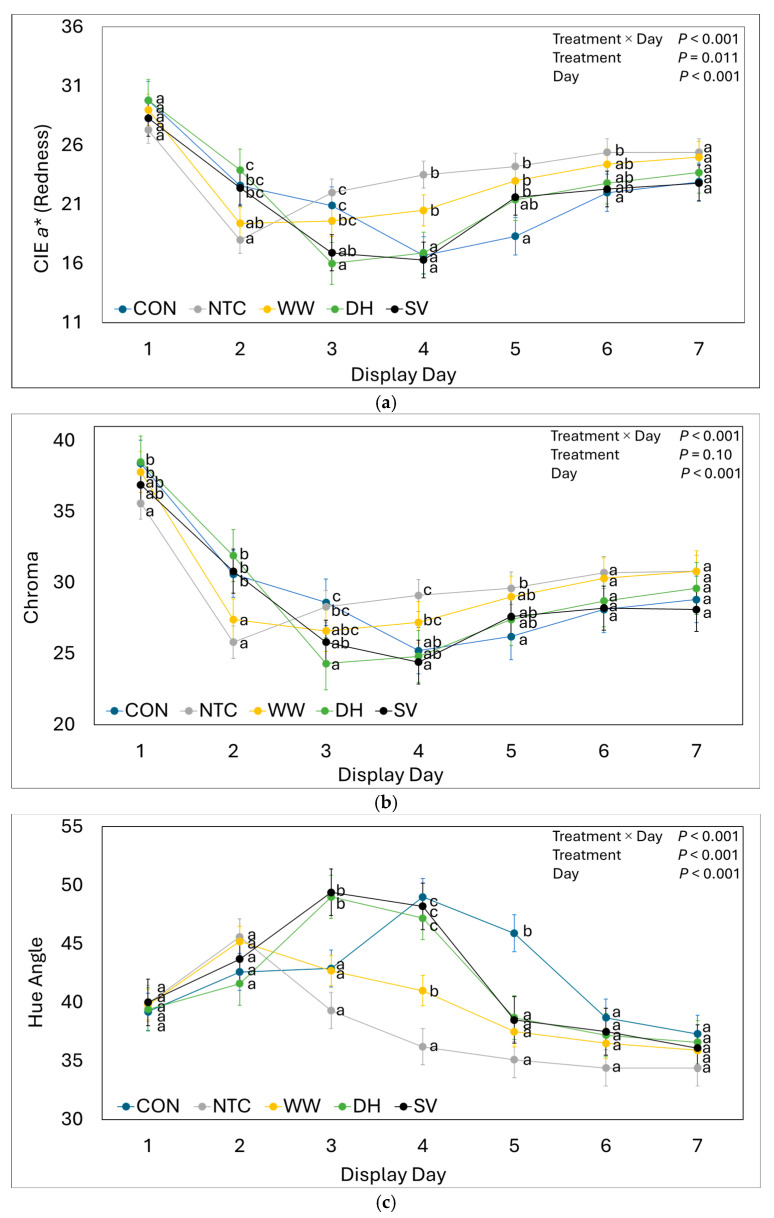
Instrumental color evaluation of ground beef patties with differently treated dry-aged crust inclusions: (**a**) CIE a*, (**b**) chroma, (**c**) hue angle. ^a–c^ Different superscript letters indicate significant differences between treatments within the same display day (*p* < 0.05). Error bars represent the standard error of means.

**Figure A1:**
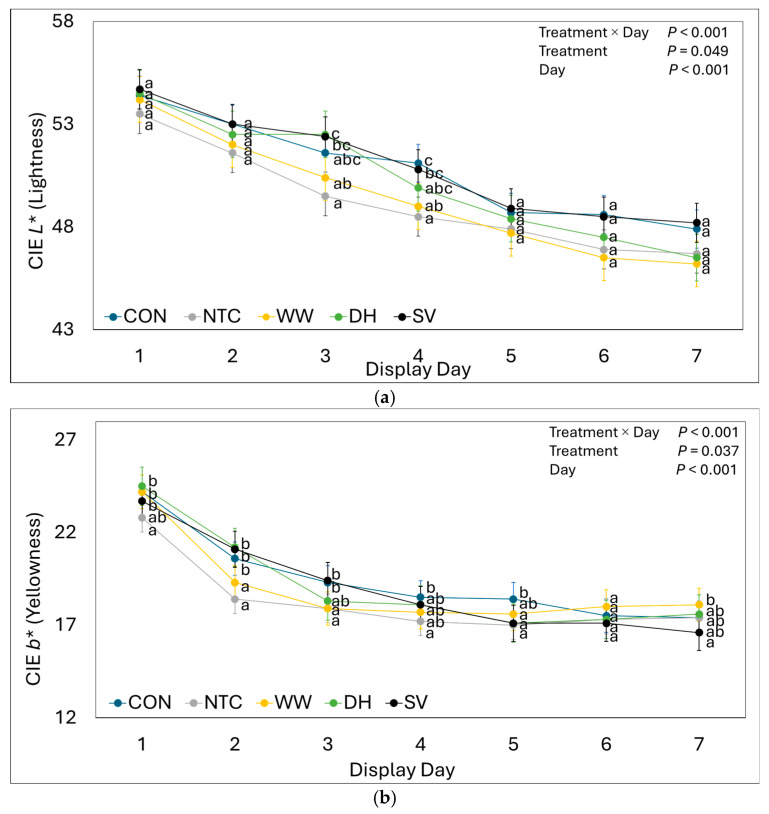
Instrumental color evaluation of ground beef patties with differently treated dry-aged crust inclusions: (**a**) CIE L*, (**b**) CIE b*. ^a–c^ Different superscript letters indicate significant differences between treatments within the same display day (*p* < 0.05). Error bars represent the standard error of means.

A significant treatment × day interaction effect was observed on all color traits measured in the current study (Figure 4 and Figure A1). Both lightness (CIE L*; Figure A1a) and yellowness (CIE b*; Figure A1b) decreased continuously throughout the simulated retail period, regardless of treatment (*p* < 0.05). While significant differences among treatments were observed within each day, differences in L* and b* values across samples were minimal throughout display and might not be practically meaningful.

Changes in redness (CIE a*; Figure 4a) were also observed across the samples throughout the display, showing an initial decline in redness, followed by a recovery in redness, regardless of treatment. Although not different on d1, a significant reduction in redness was observed on d2, with NTC exhibiting the lowest value, followed by WW, SV and CON as intermediate, and DH had the highest redness (*p* < 0.05). On d3 of display, however, NTC was observed to have a recovery in redness, showing higher redness compared to DH and SV (*p* < 0.05), where those treatments continued to decline in redness. On d4 of display, DH, SV, and CON were found to have lower redness compared to both NTC and WW (*p* < 0.05). On d5, a redness recovery pattern was observed across all samples until the end of display, where the samples showed similar redness values at the completion of display (*p* > 0.05). Chroma (Figure 4b) measurement also demonstrated a similar pattern, with the chroma value rapidly decreasing for both NTC and WW on d2 of the display compared to the other treatments (*p* < 0.05) and immediately starting to recover from d3 until the end of the display. Chroma for DH samples was observed to reach a minimum at d3 of display, while CON and SV reached the lowest level at d4, which was then followed by a color recovery until the end of display.

Hue angle (instrumental discoloration, Figure 4c) results showed the inverse of CIE a* and chroma, where the discoloration increased at the beginning of the display and disappeared by the end of the display. No differences were observed between the treatments on d1 and d2 of display (*p* > 0.05). However, NTC and WW samples showed reduced discoloration on d3 and were lower than all other treatments (*p* < 0.05). On d4, CON, SV and DH had the highest hue angle value compared to all other treatments (*p* < 0.05), and CON maintained the highest discoloration among all treatments until d5 of display (*p* < 0.05). All samples exhibited discoloration reduction on d6 and did not differ from each other until the end of display (*p* > 0.05).

The removal of moisture during dry-aging created dehydrated meat surfaces that have a darker color. The dry-aging process also generated a product with a darker color than its wet-aged counterpart, as the moisture removal reduces light reflectance [38]. The inclusion of treated dry-aged crust in beef patties in the current study did not immediately impact the initial color of the products. Nonetheless, the inclusion of treated crust altered the color quality and the color stability during the display. The current results showed a rapid color decline and discoloration development in patties containing WW and NTCs compared to SV and DH crusts. In the current study, the addition of NTC and WW crust increased the initial microbial concentrations in beef patties, whereas those with SV and DH crust additions had lower microbial concentrations, potentially due to the heat treatments applied to SV and DH crusts reducing microbial concentrations in the added crusts. Microbial growth has been well-reported to contribute to and accelerate meat discoloration [39], and thus the application of heat treatment to the dry-aged crust prior to incorporation could help reduce microbial presence and minimize negative color impact on the final beef patties due to their activity.

Interestingly, the high initial microbial concentration introduced by WW and NTC at the beginning of the display might also have a role in the faster color reversion observed in those samples in the current study. A previous study by Faustman et al. [40] reported that a very high population of psychrotropic bacteria could induce color recovery in meat homogenate. Those authors found a reduction in metmyoglobin percentage when microbial concentration reached around 10^8^ CFU and hypothesized that the recovery potentially occurred due to the interaction between myoglobin and bacterial metabolites, altering the form of myoglobin. The color recovery phenomenon has been previously reported by Smith et al. [41] and Catrett et al. [42], in which an improvement in color quality was observed during the display of ground beef and whole-muscle beef products, although no clear explanation was provided by those authors. Nevertheless, direct biochemical evaluation, such as metmyoglobin quantification and characterization of specific bacterial metabolites, is still required to definitively validate this hypothesis.

### 3.6. Thiobarbituric Acid Reactive Substance (TBARS)

**Figure 5:**
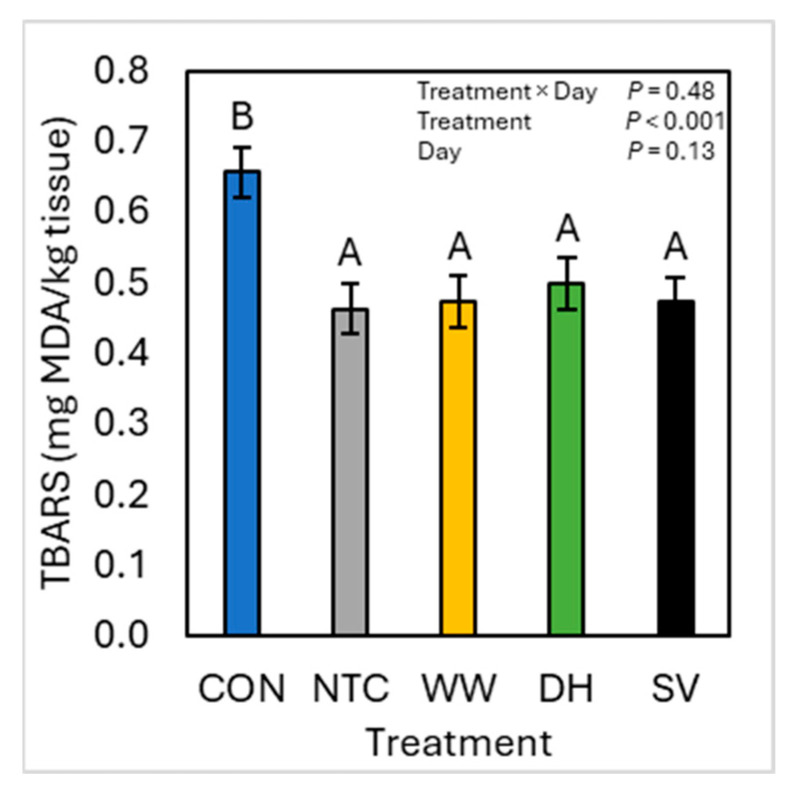
Thiobarbituric acid reactive substance (TBARS) values of ground beef patties with differently treated dry-aged crust inclusions. ^AB^ Different superscript letters indicated a significant treatment effect (*p* < 0.05). Error bars represent the standard error of means.

Significant differences in lipid oxidation were observed (*p* = 0.00196; Figure 5), with greater oxidation in CON than in other treatments. There were no significant differences found in the day (*p* = 0.127) and treatment × day interactions (*p* = 0.481).

As retail display continues, it is expected that meat will continue to oxidize. This oxidation can result in off-flavors and color changes that are not desirable to the consumer. The current study aligns with the results reported by Park et al. [10], which found decreased lipid oxidation with the inclusion of crust. These results, however, conflict with those of Xue et al. [9], in which the authors found increased lipid oxidation in the patties with the dry-aged crust inclusion. The authors explained that this might be due to increased exposure to ambient oxygen during aging, leading to greater lipid oxidation in the crust and thus influencing the final dry-aged product.

While the inclusion of crust at different levels or following different aging conditions could impact the final product differently, in this study, the inclusion of treated dry-aged crust did not increase TBARS values of the final ground beef product. A previous study suggested that dry-aged crust contains small bioactive peptides produced through endogenous and microbial proteolysis, which possess potential antioxidant properties [43]. Although not directly measured in the current study, it is possible that the dry-aged crust used in this experiment contained similar antioxidative peptides generated through the dry-aging process, which might have contributed to the lower lipid oxidation observed in patties with crust inclusions. Further investigation, however, is required to isolate and identify these specific bioactive compounds to validate this mechanism. Although there were changes in lipid oxidation levels, Campo et al. [44] reported that a TBARS value of 2.28 mg malonaldehyde/kg is the threshold at which consumers begin to notice off-flavors in beef products. All samples in this study were under this level, showing that unacceptable off-flavors after a 7 d retail display are not likely.

### 3.7. Texture Profile Analysis (TPA)

Texture profile analysis (TPA) was used to determine the structural and sensory traits of meat products. Significant differences were found in hardness (*p* = 0.006), with SV samples exhibiting the highest values compared with CON, NTC, and WW, whereas DH did not differ from any of the other treatments. The resilience (*p* = 0.008) of the CON patties was significantly higher than that of the NTC and SV patties, while WW and DH patties were intermediate and did not differ from any of the treatments. The cohesion (*p* = 0.014) of the patties showed significant differences, demonstrating a similar trend to resilience. No significant differences were found in the adhesiveness (*p* = 0.852), springiness (*p* = 0.087), and chewiness (*p* = 0.163) of the products regardless of the treatment (Table 2).

The increased hardness observed in the SV and DH samples may be attributed to the crusts being exposed to heat during preparation. It has been well reported that exposure to heat could cause the muscle structure to contract and shrink, leading to an increase in toughness [45]. It is likely that those crusts underwent thermal shrinkage during preparation, which could subsequently contribute to the increased hardness observed in the current SV and DH patties compared to those from the NTC and WW patties. Further investigation into structural shrinkage and changes in protein binding ability would be required to validate this proposed mechanism.

In general, the current study observed increased hardness when crust was added into the patties. Similar observations were reported by Xue et al. [9] in beef patties and Lee et al. [5] in pork patties. As previously mentioned, those authors added the crust in lyophilized form. Lee et al. [5] explained that lyophilized crust might absorb moisture and exhibit increased binding strength, thereby increasing patty hardness. This aligns with findings from Choi et al. [46], where they found increased binding strength with the addition of lyophilized tofu powder into ground patties. However, as minimal dehydration occurred in the crust utilized in the study compared to lyophilized crusts, the impact on hardness was less pronounced. This was also observed by Park et al. [10], where the author reported that the addition of lyophilized crust combined with water into ground beef reduced the hardness and chewiness of the patties, demonstrating that final textural properties could be heavily influenced by the hydration state of the additive and overall formulation moisture.

## 4. Conclusions

In the current study, the impact of incorporating differently treated dry-aged crust into ground beef was evaluated. The results showed that the addition of treated crust produced products with similar pH and water holding capacity. The application of dehydration and sous vide to dry-aged crust significantly reduced the overall microbial concentration of the products compared to those with untreated or warm-water-washed crust, potentially leading to better color stability in those products. The addition of the crust reduced the extent of lipid oxidation, regardless of treatment. Additionally, the inclusion of crust did not affect most of the texture attributes of the product. This indicated that dehydration and sous vide interventions have minimal impact on final product quality while reducing initial microbial concentrations, suggesting their potential use as intervention methods for dry-aged crust.

While this study provides insights into potential interventions to valorize dry-aged crust, several limitations remain, and future research is needed to facilitate their broader use. In the current study, dry-aged crusts were obtained from a single company and used at a single inclusion level. This limits the variability of the raw dry-aging crust material and constrains the implications of our study. Further studies to evaluate different dry-aged crust sources/composition and with different inclusion levels would greatly help to optimize the applicability of the proposed interventions. Future research should also focus on exploring the underlying mechanisms responsible for the antioxidative capacity measured in the dry-aged crust, pathogen evaluation in final products to confirm safety, and applying sensory evaluation to assess consumer acceptability to further validate the potential application of the crust.

## Figures and Tables

**Table 1:** Proximate composition, display loss, and cook loss values of ground beef patties with differently treated dry-aged crust inclusions.

Treatment ^1^	Moisture (%)	Ash (%)	Protein (%)	Fat ^2^ (%)	Display Loss ^3^ (%)	Cook Loss ^4^ (%)
CON	61.30	0.016	25.60	13.00	1.28	31.90
NTC	60.20	0.016	25.60	14.20	1.61	27.00
WW	60.30	0.016	26.90	12.80	1.55	28.20
DH	59.80	0.016	26.00	14.20	1.26	27.20
SV	60.00	0.016	25.80	14.10	1.85	27.80
SEM	0.79	0.0054	1.01	1.30	0.26	1.38
*p*-value	0.68	0.98	0.90	0.88	0.48	0.16

Significance was declared at *p* < 0.05. ^1^ CON = no crust inclusion; NTC = non-treated crust inclusion (10%); WW = warm water crust inclusion (10%); DH = dehydrated crust inclusion (10%); SV = sous vide crust inclusion (10%). ^2^ Fat: 100 − (Moisture + Ash + Protein). ^3^ Display loss: [{(d1 wt. − d7 wt.)/(d1 wt.)} × 100]. ^4^ Cook loss: [{(raw wt. − cooked wt.)/(raw wt.)} × 100]. SEM: standard error of means.

**Table 2:** Textural properties of cooked ground beef patties with differently treated dry-aged crust inclusions.

Treatment ^1^	Hardness (g)	Adhesiveness (g.s)	Resilience (%)	Cohesion (%)	Springiness (%)	Chewiness
CON	9926 ^A^	0.15	8.04 ^B^	0.24 ^B^	23.60	567
NTC	11,023 ^A^	0.17	4.67 ^A^	0.16 ^A^	17.30	312
WW	10,950 ^A^	0.21	6.56 ^AB^	0.21 ^AB^	22.00	519
DH	12,503 ^AB^	0.27	5.56 ^AB^	0.19 ^AB^	19.90	463
SV	14,551 ^B^	0.17	4.18 ^A^	0.15 ^A^	14.50	306
SEM	688	0.08	0.62	0.017	2.14	82.20
*p*-value	0.0060	0.85	0.010	0.014	0.090	0.16

^AB^ Means within a column with different superscripts are significantly different (*p* < 0.05). ^1^ CON = no crust inclusion; NTC = non-treated crust inclusion (10%); WW = warm water crust inclusion (10%); DH = dehydrated crust inclusion (10%); SV = sous vide crust inclusion (10%). SEM: standard error of means.

## Data Availability

The original contributions presented in this study are included in the article. Further inquiries can be directed to the corresponding author.

## Abbreviations

The following abbreviations are used in this manuscript:

d1	Day 1
d7	Day 7
CON	Control; no crust inclusion
NTC	Non-treated crust
WW	Warm water wash
DH	Dehydrated
SV	Sous-vide
APC	Aerobic plate count
LAB	Lactic acid bacteria
YM	Yeast/mold
TBARS	Thiobarbituric acid reactive substances
TPA	Texture profile analysis
RCBD	Randomized complete block design
